# Tinnitus with a normal audiogram: Role of high-frequency sensitivity and reanalysis of brainstem-response measures to avoid audiometric over-matching

**DOI:** 10.1016/j.heares.2017.10.002

**Published:** 2017-12

**Authors:** Hannah Guest, Kevin J. Munro, Christopher J. Plack

**Affiliations:** Manchester Centre for Audiology and Deafness, University of Manchester, Manchester Academic Health Science Centre, UK; NIHR Manchester Biomedical Research Centre, Central Manchester University Hospitals NHS Foundation Trust, Manchester Academic Health Science Centre, UK; Manchester Centre for Audiology and Deafness, University of Manchester, Manchester Academic Health Science Centre, UK; NIHR Manchester Biomedical Research Centre, Central Manchester University Hospitals NHS Foundation Trust, Manchester Academic Health Science Centre, UK; Department of Psychology, Lancaster University, Lancaster, UK

**Keywords:** ABR, auditory brainstem response, EHF, extended high-frequency, EFR, envelope-following response, SEM, standard error of the mean

In Guest et al. (2017), we tested for associations between tinnitus and electrophysiological measures of cochlear synaptopathy in young humans with normal hearing sensitivity. Tinnitus and control groups were matched closely for age, sex, and audiometric thresholds up to 14 kHz. The groups did not differ significantly in auditory-brainstem-response (ABR) or envelope-following-response (EFR) measures of synaptopathy.

The matching of audiograms at extended high frequencies (EHFs) was intended to prevent confounding effects of EHF audiometric loss on brainstem-response measures. Such effects are, in our view, a potential pitfall in synaptopathy research, which tends to employ high stimulus levels that likely elicit contributions from the extreme cochlear base (for example, 120 dB pSPL in Gu et al., 2012; 130 dB peSPL in Liberman et al., 2016). Derived-band responses in humans indicate that ABR wave I is dominated by high-frequency generators, including those above 8 kHz (Don and Eggermont, 1978, Hardy et al., 2017), and increasingly so at high stimulus levels (Eggermont and Don, 1980). Hardy et al. (2017; personal communication, 10/02/17) recently demonstrated that both wave I amplitude and the ratio of wave I amplitude to wave V amplitude are reduced when noise high-pass filtered at 8 kHz is added to remove contributions from EHF regions. Their findings raise questions about apparent evidence for cochlear synaptopathy in humans, since such evidence has often been accompanied by EHF audiometric deficits (Gu et al., 2012, Liberman et al., 2016, Schaette and McAlpine, 2011), or even deficits at standard audiometric frequencies (Bramhall et al., 2017).

However, it has come to our attention that control of audiometric factors in our tinnitus study might have come at a cost. Hickox et al. (2017) note that many animal models of synaptopathy additionally produce some degree of basal hair-cell loss. Liberman et al. (2016) posit that “high-frequency threshold elevation will be correlated with mid-frequency cochlear synaptopathy”. If this expectation is justified, then over-matching of audiometric thresholds in our study might have risked obscuring genuine differences in auditory nerve function between groups. Future research might usefully address this issue by allowing variation in EHF audiometric thresholds and preventing their direct influence on proxy measures of synaptopathy through the application of high-pass masking (Hardy et al., 2017, Hickox et al., 2017, Liberman et al., 2016).

Though we did not adopt this approach in our study, we reasoned that reanalysis without EHF matching might shed new light on our findings. Our decision to match thresholds up to 14 kHz may have been overzealous, since our stimuli possessed a narrower bandwidth than those of some previous studies (Gu et al., 2012, Schaette and McAlpine, 2011) and a far lower level than one study (Gu et al., 2012). The combination of restricted bandwidth, moderate stimulus level, and audiometric matching (to within 1 dB at 14 kHz) may have represented an excessively cautious approach.

Therefore, we repeated our original ABR and EFR analyses with groups matched solely for age and sex. Two participants were added to the tinnitus group (both female, with prolonged spontaneous tinnitus of >15 years duration) and the resulting 22 participants were matched with 22 controls drawn from a reservoir of 41 potential matches. This reservoir was composed of our original control group plus controls from a later study investigating listening difficulties and synaptopathy, whose measures encompassed those employed in the tinnitus study. Selection of controls was conducted via optimal pair matching using the “optmatch” *R* package (Hansen and Klopfer, 2006). Recruitment of tinnitus and control participants was based on normal pure-tone audiometry between 0.25 and 8 kHz, normal middle ear function, normal otological history, and age (18–40 years), but was otherwise unrestricted. Although we can't discount possible biases related to participants' willingness to participate, we consider that these groups are essentially a random sample of normal-hearing people with and without tinnitus in this age range.

**Fig. 1:**
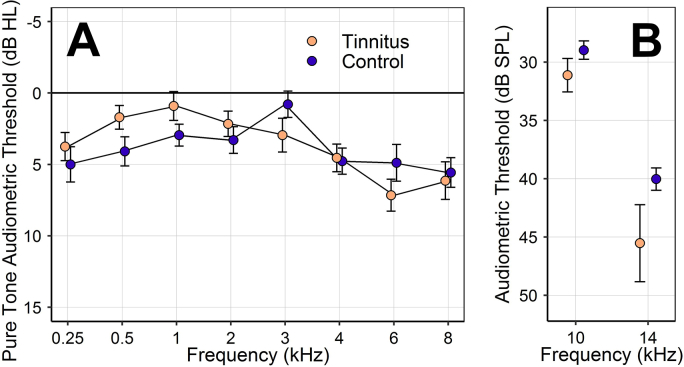
Mean audiometric thresholds for the tinnitus and control groups. Error bars represent the standard error of the mean (SEM). **A**: Pure-tone audiometric thresholds. **B**: EHF audiometric thresholds for 1/3-octave narrowband noise.

**Fig. 2:**
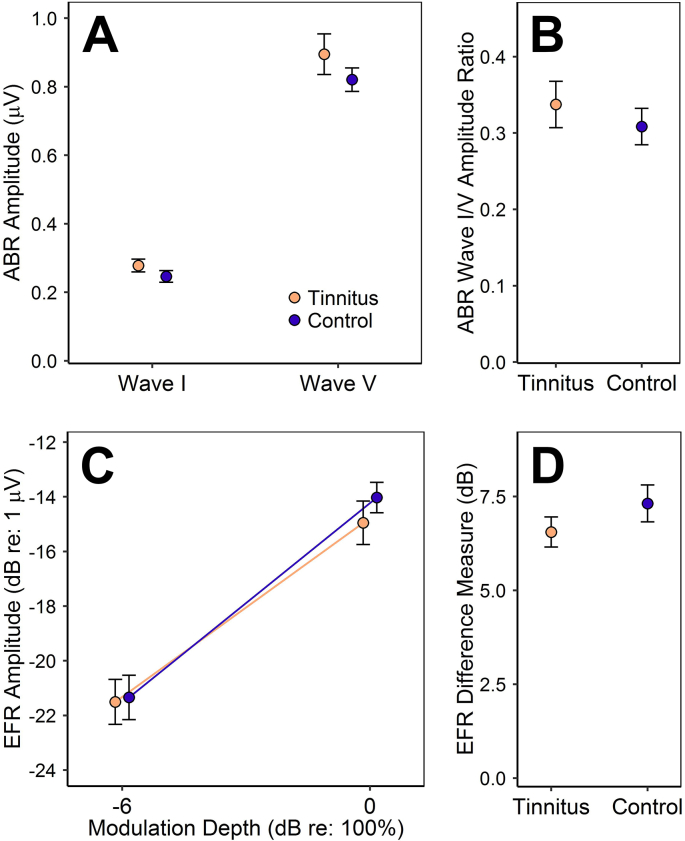
Brainstem-response measures of synaptopathy for the tinnitus and control groups. Points and error bars represent mean ± SEM. **A**: The amplitudes of ABR wave I and wave V. **B**: The ratio of ABR wave I amplitude to wave V amplitude. **C**: The amplitudes of EFRs to stimuli of differing modulation depths: shallow (−6 dB) and full (0 dB). **D**: The difference in EFR amplitude at the two modulation depths. Note that this measure is expected to *increase* in ears with preferential loss of high-threshold fibers.

The resulting groups are each 55% female and have similar mean ages (tinnitus 26.6 years, control 26.5 years), but differ substantially in EHF sensitivity (Fig. 1). Group comparisons of ABR and EFR measures of synaptopathy reveal no significant associations with tinnitus, just as in the original analyses (Fig. 2). This is true of both raw amplitude measures and self-normalized difference measures: *p* > 0.23 (two-tailed) in all cases, as determined by independent-samples *t*-tests and mixed two-way ANOVA.

Hence, we find no indication that the null results of our study were a consequence of audiometric over-matching. Our original conclusion stands, namely that we find no evidence for cochlear synaptopathy in tinnitus with a normal audiogram. The results also suggest that our ABRs and EFRs were not substantially affected by EHF audiometric function, presumably due to the combination of restricted stimulus bandwidth and relatively low presentation level. However, we caution that this may not be true of other ABR and EFR measures, and that careful control of EHF contributions should be a priority in synaptopathy research. Without such efforts, it will not be possible to establish whether associations between EHF audiometric loss and electrophysiological measures are due to direct causal effects or – as in the view of Liberman et al. (2016) – to correlations between EHF loss and synaptopathy. In short, a crucial aim of future research must be to discern whether EHF audiometric loss is a *marker* or a *mimic* of cochlear synaptopathy.
